# The developmental path to gambling disorder among U.S. veterans: a life-course perspective

**DOI:** 10.3389/fpsyg.2026.1766736

**Published:** 2026-06-15

**Authors:** Belle Gavriel-Fried, Naama Glaser-Guy, Jenia Kelner, Karen A. Valle Frias, Shane W. Kraus

**Affiliations:** 1 The Bob Shapell School of Social Work, Tel Aviv University, Tel Aviv, Israel; 2 Department of Psychology, University of Nevada Las Vegas, Las Vegas, NV, United States

**Keywords:** gambling disorder, pathways model, life-course perspective, risk factors, U.S. veterans

## Abstract

**Introduction:**

Gambling disorder (GD) is characterized by persistent gambling behavior associated with significant distress and functional impairment. Pathways to GD reflect a complex interplay of individual, environmental, and experiential factors. United States (U.S.) veterans are considered particularly vulnerable due to heightened exposure to trauma and cumulative life stressors. In a qualitative study examining barriers to treatment among veterans, participants described various risk factors contributing to GD, prompting a secondary analysis to better identify the unique risk factors shaping the pathways leading U.S. military veterans and service members to develop and persist in GD.

**Methods:**

Participants included 28 U.S. service members and veterans (8 women; aged 27–73, *M*_age_ = 46.5) recruited in the primary study. Semi-structured interviews were conducted via Zoom and included an assessment of GD diagnostic criteria (past year and 10-year history). A combination of secondary analysis and a theory-informed approach was employed, using the Pathways Model of Problem and Pathological Gambling as the overarching theoretical framework. In practice, the analysis combined deductive and inductive approaches to content analysis.

**Results:**

Three main themes were revealed, each representing a specific life period: childhood, military service, and civilian life. The themes included: a) life history of vulnerability and yearning for change; b) the broken promise: the military as a multidimensional risk for developing gambling problems; and c) sinking deeper into gambling in the post-military period. While these themes corresponded with the second subtype of the Pathways Model—emotional vulnerability—they primarily illustrate the dynamic interplay between individual vulnerability and the changing socio-cultural and environmental contexts across the participants’ life stages and turning points.

**Discussion:**

Integrating the Pathways Model with a life-course perspective, findings underscore the dynamic interplay between individual vulnerability and environmental context in shaping GD trajectories across veterans’ life stages. Rather than disrupting risk, key transitions may reinforce maladaptive patterns over time. Future studies should incorporate a life-course perspective into the theoretical framework of the Pathways Model of problem gambling.

## Introduction

This study brings together two research domains: the risk factors that constitute the pathways leading to gambling disorder (GD) and the experiences of United States (U.S.) military veterans living with GD. While each of these topics has been extensively explored, there remains a significant gap in the gambling literature. The traditional literature has predominantly focused on clinical samples (Excell et al., 2022), with limited attention to military veterans’ life experiences. To address this gap, the current study aimed to identify the specific risk factors that shape the pathways through which GD develops and persists among veterans.

GD is defined as a pattern of persistent gambling behavior that often leads to distress and functional impairments in domains such as social, emotional, and personal (American Psychiatric Association, 2022). While GD refers to the clinical diagnosis of gambling behaviors, the term problem gambling (PrG) is often used to describe a subclinical group of individuals who experience similar levels of distress as those with GD but do not yet meet the full diagnostic criteria for the disorder (King and Wong-Padoongpatt, 2023).

Precise prevalence estimates of GD among American adults remain elusive, though rates are generally estimated to be below 1%, whereas PrG rates vary between 2 and 3% (American Psychiatric Association, 2013; Tran et al., 2024). In general, men experience higher PrG rates than women, and younger adults are at greater risk for PrG than older adults (>70 years) (Afifi et al., 2010; Tran et al., 2024).

Recent studies have also identified military veterans as a particularly vulnerable group for developing PrG compared to the general adult population (Dighton et al., 2023; Etuk et al., 2020; Metcalf et al., 2022). In a previous study on treatment barriers among veterans (Vana et al., 2025), participants described various factors contributing to GD.

Among non-military samples, scholars have noted several pathways to PrG, often reflecting a complex interplay between individual, environmental, and gambling-specific factors (Oksanen et al., 2021; Nower et al., 2022b). Multiple theories, models, and empirical studies have sought to explain the mechanisms underlying the development of PrG. *The Social Ecological Theory of Problem Gambling* emphasizes how gambling behaviors are influenced by different levels of the environment (Oksanen et al., 2021). The model identifies spheres that combine individual and environmental factors, such as age, personality, and their interactions with interpersonal, organizational, and societal influences to explain PrG (Oksanen et al., 2021). This model has also been applied cross-nationally to adolescents and young adults from the U.S., South Korea, Spain, and Finland, revealing variations in PrG across countries. These variations were explained by the organizational sphere (e.g., online casinos, online gambling communities, exposure to online advertisements, etc.), as this sphere had strong associations with PrG (Oksanen et al., 2021).

The bio-psychosocial model views addictions, including PrG, as the result of interactions between biological and psychological factors (Buchman et al., 2010; Sharpe, 2002; Wangensteen and Hystad, 2022). Specifically, environmental and interpersonal factors have also been linked to PrG and GD. These include having a lower quality of life (e.g., low education, unemployment, etc.) and having family members who also gamble (Vintró-Alcaraz et al., 2022; Nower et al., 2022a). Other risk factors include low social support, heightened trait impulsivity, severity of gambling-related cognitive distortions, and the legalization of gambling avenues (Mancini, 2025; Mestre-Bach et al., 2021; Vintró-Alcaraz et al., 2022; Yeola et al., 2025).

Past research has described GD as a progressive disorder leading to increasingly severe consequences over time. A recent review of longitudinal studies suggests that GD is better understood as an “enduring condition,” which may remit and relapse over time (Williams and Williams, 2025). Specifically, this review identified key contributing factors such as gambling fallacies, social gambling influences, addiction propensity, impulsivity, and intensive gambling involvement (Williams and Williams, 2025). Similarly, a longitudinal study of 2,018 Australian men found that low social support predicted problem gambling severity and frequency (Mancini, 2025). Another study found that adverse childhood experiences were linked to an increased risk of more severe pathological gambling (Lotzin et al., 2018).

The seminal *Pathways Model* (Blaszczynski and Nower, 2002) and its revised version (Nower et al., 2022b) identified three subgroups of problem gamblers: (a) behavioral conditioned gamblers—those who gamble for social and recreational reasons and are reinforced through wins; (b) those who gamble to escape negative mood states and often have adverse life experiences; and (c) antisocial impulsivist gamblers—those characterized by antisocial traits and high impulsivity who gamble to cope with stress. All three pathways integrate biological, personality, developmental, cognitive, learning, and ecological factors, emphasizing that accessibility and availability of gambling are foundational influences (Nower et al., 2022b). The revised version of the Pathways Model maintained the three pathways originally proposed but also provided evidence that Pathway 2 (emotionally vulnerable) and Pathway 3 (antisocial impulsivist) are independent, rather than treating Pathway 3 as a subgroup of Pathway 2 (Nower et al., 2022b). Various studies have empirically tested the Pathways Model in various populations. For instance, researchers (Bonnaire et al., 2022) found that emotional dysregulation, cognitive biases, and impulsivity predicted PrG among treatment seeking gamblers in France. Similarly, a longitudinal study of Finnish adults found that gambling motives—winning money, competition, and escape—predicted gambling problems, consistent with the Pathways Model (Hagfors et al., 2023).

Presently, veterans represent a high-risk group for GD. Specific risk factors such as individual characteristics, psychiatric comorbidity, military cultural norms, and environmental and financial stressors during and after military service contribute to this vulnerability. For example, one study found that 70% of U.S. veterans receiving inpatient treatment for GD met diagnostic criteria for at least one other psychiatric disorder (Grubbs et al., 2023); in general, among the military population, GD commonly occurs alongside substance use, anxiety, mood disorders, or nicotine dependence (Dowling et al., 2015). Suicidality (i.e., ideation and attempts) is particularly elevated among veterans with GD (Valle Frias et al., 2025).

*Ecological and organizational* factors also heighten risk for U.S. service members. For example, these include military roles involving boredom or downtime and barriers to treatment such as stigma, limited treatment resources, and fear of repercussions (Champion et al., 2025; Vana et al., 2025). Another prominent risk factor is access to slot machines on overseas U.S. bases. A report written to Congress by the Government Accountability Office (GAO) found that the Department of Defense’s slot machines on U.S. overseas bases generated over $500 million in revenue from 2011 to 2015 (U.S. Government Accountability Office, 2017), demonstrating the high accessibility of gambling and usage among service members.

In addition, *societal* factors such as increased accessibility to gambling avenues for veterans transitioning to civilian life also contribute to the risk of PrG. These include the recent legalization of sports betting in the U.S. (Yeola et al., 2025), family stress, low marital quality (Pflieger et al., 2018), and limited GD treatment options within the Department of Veterans Affairs (VA) (Vana et al., 2025); we posit that all of these factors collectively increase U.S. veterans’ vulnerability for GD.

Research has examined the hardships that U.S. service members face when being discharged from the military, which make them vulnerable to PrG. Some discharged service members experience psychiatric diagnoses such as PTSD, with lifetime prevalence among US adults being 6% for civilians and 7% for veterans (Chambliss et al., 2024). Among veterans with GD, over 25% report chronic homelessness, often associated with mental illness, trauma, suicidality, and incarceration (Stefanovics et al., 2023). A study on veterans after deployment found more severe gambling problems in males with low social support and stressful life events post deployment (Whiting et al., 2016). In addition, Australian veterans with gambling problems who recently transitioned to civilian life exhibited greater suicidality (Metcalf et al., 2023).

While most prior research on veterans and GD has been quantitative, emphasizing associated risk factors and largely based on treatment-seeking GD patients, few studies have specifically explored the lived experiences of veterans and service members with GD. In a study aiming to identify the internal and external barriers to treatment among military veterans and service members, hereafter referred to as the “primary study” (Vana et al., 2025), participants often shared stressful life stories and environmental risks that reflected the events and factors leading them to develop GD. These insights guided our decision to deepen our analysis of the existing data from the primary study and apply the underlying concept of the Pathways Model of PrG (Blaszczynski and Nower, 2002; Nower et al., 2022b) as a possible theoretical framework to identify risk factors associated with the development of GD that shape the pathways to GD among military populations. Hence, the current study seeks to identify the unique risk factors shaping the pathways through which U.S. military members develop and persist in GD, based on their lived experiences and the specific nuances of these pathways within this group.

## Methods

### Methodological approach

In the present study, we employed two complementary approaches: a secondary qualitative data analysis (Heaton, 2008), and a theory-informed retrospective approach (Giles and Harrison, 2023). Secondary qualitative data analysis is typically conducted to address a research question that differs from that of the primary study, allowing for the exploration of new theoretical ideas and deeper insights (Heaton, 2008; Ruggiano and Perry, 2019). Following the principles of a theory-informed approach (Giles and Harrison, 2023), we used the Pathways Model of problem gambling (Blaszczynski and Nower, 2002; Nower et al., 2022b), which provided a theoretical framework to formulate our research question, select relevant data, and interpret our findings. We found these two complementary approaches particularly suitable for our research aim: to explore the risk factors that shape and construct pathways through which veterans develop and persist in GD—an aspect that was not explored in the primary study. Utilizing these methodological approaches enables a deeper understanding of the interviews collected in the primary study and helps generate new theoretical meanings.

### Participants and procedure

In the primary study (Vana et al., 2025), 28 participants (8 women) aged 27–73 years (*M*__age_ = 46.5) were recruited. The sample included 24 veterans and 4 active service members. Nine participants were married or partnered. Regarding race/ethnicity, 15 identified as White, 6 as Black, and 7 as Latino, Asian, Native American, or Multiracial. Branch representation included 10 Air Force, 5 Navy, 4 Marine Corps, and 9 Army participants.

U.S. veterans and service members were recruited utilizing a purposive sample that combined maximum variation and criterion-based case selection strategies (Patton, 2014). Eligibility was limited to individuals who were at least 18 years of age, currently serving in or formally discharged from the U.S. Armed Forces, and those who currently met the diagnosis for GD or had met the GD diagnosis in the past 10 years, as determined by endorsing at least four symptoms of the GD criteria outlined in the DSM-5 (American Psychiatric Association, 2013). Of the 28 participants, 22 currently met the diagnostic criteria for GD (non-recovered), while 6 participants (2 Army, 4 Air Force) were in remission (recovered). Of the six in remission, 5 were veterans and 1 was a service member (Air Force).

Participants were recruited nationwide through flyers, social media platforms, outreach to organizations supporting veterans, and engagement with individuals working in veteran communities. Recruitment continued until sufficient data saturation was achieved for the primary study (Vana et al., 2025).

Eligibility was determined via a structured screening conducted over Zoom by a clinical psychology doctoral student. A standardized self-report protocol was administered to assess: (a) informed consent, (b) demographic characteristics, (c) clinical inclusion criteria—specifically, meeting DSM-5 criteria for GD within the past 10 years, including both current GD (past 12 months) and remission status (i.e., individuals who met GD criteria within the past 10 years but not within the past 12 months). Individuals were excluded if they reported any other active addictions.

All initial screenings (approximately 15–20 min) and subsequent in-depth interviews (approximately 60–90 min) were supervised by a licensed clinical psychologist who provided weekly face-to-face supervision and feedback on all completed screens and interviews. Electronic field notes were completed immediately after each interview, accessed during transcription and reviewed by the research team during data analysis.

Interviews were video recorded (with audio) and transcribed verbatim. Participants were asked open-ended questions designed to elicit detailed accounts of their experiences with gambling problems across their life course, including before, during, and after military service. Example prompts included: “Tell me about yourself as a veteran who has experienced problems with gambling” and “Do you think any aspect of your military service contributed to your gambling problems, or do you view them as separate?”

Participants received a $75 Advarra card upon completion. Data collection occurred between August 2023 and August 2024. The authors’ Institutional Review Board approved the study, and all participant names are pseudonyms to protect confidentiality.

### Data analysis

The analysis combined conventional (inductive) and directed (deductive) content analyses (Hsieh and Shannon, 2005). This approach allowed us to interpret the findings through the theoretical lens of the Pathways Model (Blaszczynski and Nower, 2002; Nower et al., 2022b) while also remaining open to new insights related to various risk factors emerging from the veterans’ accounts. The process unfolded in several steps: In the first phase, the new team members (N. G. G and J. K) who were not involved in the primary study immersed themselves in the interviews. During this stage, they noted initial impressions, recurring ideas, and emerging patterns related to gambling motives, experiences, and contexts. This phase was intentionally inductive, allowing ideas to surface naturally from participants’ narratives. A main theme related to factors leading to GD prompted us to utilize various known risk factors, specifically the Pathways Model in problem gambling (Blaszczynski and Nower, 2002; Nower et al., 2022b), to formulate our research question, analyze the interviews, and interpret the results.

Then, five interviews were coded together by the same two team members, integrating deductive and inductive coding approaches. The deductive approach was based on various known risk factors and specifically the determinants of the Pathways Model in problem gambling (e.g., ecological, personal, emotional vulnerability) (Blaszczynski and Nower, 2002; Nower et al., 2022b). To explore the nuances related to veterans’ pathways, an inductive coding approach was utilized, including all risk factors related to the military mentioned by the interviewees. This prevented bias toward the Pathways Model. After a discussion with the first author about the codes revealed from the five interviews, these codes were refined, and the two team members coded the remaining 23 interviews separately, using the codes that had already been identified (e.g., history of sexual violence; alcoholic parents; stress and challenges in the military) while also inductively identifying new codes. During this stage, the three team members (B. G. G, N. G. G, J. K) subsequently met to compare and critically discuss the coding. Discrepancies were examined through an iterative, reflexive process until consensus was reached. In most cases, there was substantial agreement between coders. At the end of this stage, 64 codes were identified (e.g., stress and challenges in the military, gambling in the family, gambling out of boredom, gambling with other veterans, gambling started because there are machines on base). At the next stage, the codes were critically discussed by the first three authors (B. G. F, N. G. G, J. K) and merged into 49 codes, which were grouped into 10 categories according to similarities and differences (e.g., emotional stressors in the military, starting points of gambling, transitions into civilian life related to gambling). These categories were merged and conceptualized into three themes that were organized according to life periods from childhood to military service to civilian life. Table 1 illustrates this analysis process. Finally, the themes were contextualized within pertinent theoretical and empirical frameworks to achieve a comprehensive understanding of veterans’ paths to GD while maintaining analytical rigor (Denzin, 2001). Other group members (SWK and KFV) critically read the findings chapter and approved it. These stages of analysis reflect an interactive process for achieving inter-rater consistency rather than quantifying agreement (Hemmler et al., 2022).

**Table 1:** Illustration of the theme, categories, and codes.

Themes	Categories	Codes
Life history of vulnerability and yearning for change	Adverse life events	History of death in the family; coping with life-threatening medical conditions; medical malpractice; divorce as a catalyst for gambling; history of sexual violence; alcoholic parents; trouble while growing up; feeling of not belonging
	Start point of gambling at early life period	Gambling starts in the family; gambling starts as a kid
	Motivations to join the military	As a reset button for gambling; as a way to gain security and future importunities; family recommendation to join; dealing with childhood issues
The broken promise: the military as a multidimensional risk for developing gambling problems	Gambling culture in the military	Gambling together with other soldiers; getting others into gambling in the military; gambling as a way to make friends in the military
	Emotional stressors and trauma in the military	Gambling out of boredom; feeling of inadequacy; loneliness in the service; hating military job; isolation as part of marginalization; gambling as a way to deal with lack of control; stress and challenges in the military; sexual trauma in the military; stress due to health issues; stress due to military culture about sexual orientation; coping with the job; assault during military service; gambling to deal with trauma in the military; gambling to get a rush
	Environmental factors in the military related to gambling	Gambling starts because there are machines on base; stationed in a place that is accessible to gambling; gambling as a solution to get out of bad housing; being female in a male environment; not making enough money in the service; receiving money and being on your own
	Starting point of gambling in the military	Gambling starts in the military; receiving free things while gambling
	The military as a protective factor from gambling	The military as a protective factor; taking protective measures during service; holding back on gambling because of the need for focus during deployment
Sinking deeper into gambling in the post-military period	Transition into civilian life related to gambling	Financial difficulties in the transition; transition into civilian life as stressful; gambling to deal with military trauma and PTSD after release; VA environment; moving after release as catalyst
	Veterans as a community of gamblers	Community of gambling veterans; gambling with other veterans

VA, Veterans Affairs.

### Trustworthiness of the analysis

Several strategies were employed to ensure the trustworthiness of the study (Guba, 1981; Lincoln and Guba, 1986). Credibility was further strengthened through close engagement with the data, including multiple readings of interview transcripts. To enhance the dependability and confirmability of the findings, the research team also engaged in ongoing collaboration, which included regular meetings to discuss and refine codes, categories, and themes, thereby maintaining analytic consistency. The use of MAXQDA software provided an organized and systematic framework for handling transcripts, codes, memos, and thematic structures, which contributed to the transferability. Finally, participants’ perspectives were foregrounded throughout the analysis by incorporating extensive direct quotations, ensuring that the findings faithfully represent their voices.

## Findings

Data analysis identified three main themes, each corresponding to a distinct period in the participants’ lives. Across all themes, personal vulnerability, stressors, and socio-cultural and environmental factors were found to contribute to the development of gambling problems. These were interpreted through the general ideas of the Pathway Model to PrG (Blaszczynski and Nower, 2002; Nower et al., 2022b) and other current research. The first theme, *Life history of vulnerability and yearning for change*, relates to the period before military service. Specifically, it highlights how adverse life experiences and their consequences, along with an environment characterized by limited opportunities, fostered participants’ early vulnerability to gambling. The second theme, *The broken promise: the military as a multidimensional risk for developing gambling problems*, focuses on how trauma and a “gambling-friendly environment” during service further increased vulnerability to developing GD. The third theme, *Sinking deeper into gambling in the post-military period*, centers on the challenges of reintegration into civilian society. Vulnerability in this phase was linked to the stress of transitioning into civilian life and unresolved trauma, including a lack of support systems and a social network in which gambling was perceived as a common behavior. These themes illustrate the dynamic interplay between individual vulnerability and the changing environmental context across the participants’ life stages. This interaction appears to shape the pathway to GD within the unique life trajectories of military veterans.

### Life history of vulnerability and yearning for change

While describing their perceptions and experiences regarding their GD, participants reflected on their past, particularly the initial exposures and formative events they saw as contributing to its development. Several participants identified childhood experiences and environmental determinants as shaping their trajectories into GD. The participants spontaneously described ecological factors such as early exposure to gambling within family contexts. Although the original Pathway Model addresses ecological factors such as accessibility and availability in relation to policy and regulatory legislation (Blaszczynski and Nower, 2002), this research suggests that the normalization and acceptability of gambling within family culture represent a more proximal ecological determinant. In addition, some participants described exposure through significant others, particularly parents, which reflects processes consistent with social learning (Blaszczynski and Nower, 2002). These experiences appear to function as early conditioning mechanisms, reinforcing gambling as both an acceptable behavior and, in some cases, a means of integration into family culture.

Brayden (57, male veteran, White, non-recovered) describes a culture of gambling in his family as something they did regularly, involving him in gambling practices as a child:

I can actually go back to when I was a kid umm our family gatherings weren't just sitting around […] We had to break the cards out to play poker. It wasn't, and you know. That's how both sides of my family were. I mean, everybody was, you know, *in the gambling kind of a hiatus*, you know. I can remember when *I was like 7 years old I learned how to play this game called cribbage,* and I used to watch my dad and uncle play for hours and they used to play for money.

Research has shown that through processes of social learning, having a gambling role model within the family environment and culture is an important risk factor for developing future gambling problems (Delfabbro and Thrupp, 2003; Dowling et al., 2021; Walters, 2021). This can be understood through learned cognitive schemas. For example, these two participants described repeatedly witnessing adult family members engage in gambling, a pattern that may contribute to the development of cognitive distortions and irrational beliefs (e.g., overestimation of one’s personal ability to win) (Toneatto, 1999).

Through the interviews, we recognized that throughout their childhood, the participants described not only exposure, availability, and acceptability of gambling through family members, but also adverse life events and vulnerabilities regarding their families and past experiences. GD is often associated with adverse life events (Estevez et al., 2023; Lotzin et al., 2018; Wang et al., 2020), creating a vulnerable foundation as part of the second pathway into GD, which identifies people who gamble as a primary function of coping with emotional distress often rooted in adverse life events (Nower et al., 2022b).

For example, Drake (72, male veteran, White, non-recovered) described an interesting route of exposure to addiction early in life, attempting to escape this path only to find that his past made him especially vulnerable to becoming addicted to gambling:

My parents were both alcoholics and fought a lot. So, and then, you know, by the time I was in fifth grade I’d already sworn off gambling- or drinking, because of my parents drinking […] Later on, I learned about action from gambling. I said, oh my god, I've been doing that since I could walk.

Peter (64, male veteran, White, non-recovered) also demonstrated the idea of early social learning and irrational cognitive and emotional processes involved in GD (Blaszczynski and Nower, 2002; Nower et al., 2022b) through his complicated relationship with his father. He described how his father exposed him to gambling as a teenager when he took him to Las Vegas in a kind of apprenticeship for gambling. He also noted how, despite witnessing the destructive impact of gambling on his father, he found himself embracing it as a coping method: “You know, I thought that I would have stayed as far away from gambling as possible, but…it’s almost like I embraced it because it’s my only way out, you know, when I don’t…want to think-think of things, you know.”

Though not always directly connected to their gambling, the participants described events that are highly correlated with the onset of GD later in life (Estevez et al., 2023; Lotzin et al., 2018; Wang et al., 2020). For example, Mark (51, male veteran, White, non-recovered) recounted his experiences as a child, feeling unheard and misunderstood by his parents:

I never fit in and nobody wanted to hear any of my problems. And if you did have problems, you can’t talk about them anyways. So, you know, that was me and my dad, and he never, he never talked about what happened over there [while growing up]. And I did try to tell my mom about stuff from when I was kid. She said, well, sometimes you need to move on, you know. Forget about it and move on. Go ahead. Don't think you can't.' You know.

This reflects both the notion of childhood maltreatment (Nower et al., 2022b) and suggests that his family’s disregard for his troubles prevented him from learning adaptive coping skills.

Ryan (73, male veteran, Multiracial, non-recovered) shared an incident of sexual assault in childhood. Previous research has found that PrG among US veterans is associated with past sexual trauma (Stefanovics et al., 2017), which could be understood as a vulnerable starting point within the second pathway. This participant described the event as significant, although he expressed uncertainty about its direct connection to gambling:

And then there were incidents during the seminary youth that I was accosted sexually by two priests and a brother and I-I have talked about all this and therapy over the years. I don't know if that's important to talk about now, but I mean when it all comes out in the wash, it's part of what happened to my life, you know, and maybe that I don't know if that has anything any bearing on the gambling. I don't know.

An interesting connection can be found between the pathway into gambling, especially among those with childhood difficulties, and their decision to join the military. Prior research suggests that joining the military is a known way to cope with a difficult childhood environment and even enhance opportunities for a better future (Helmus et al., 2018). Just as the second pathway recognizes gambling as a coping mechanism for specific vulnerabilities, this could be interpreted as a more adaptive attempt to cope with past experiences. Some participants shared that their motivation to join the military was an act of hope to change their lives for a better future in terms of finances, education, and overall quality of life. For example, Sherry (39, female veteran, Black, non-recovered) explained how joining the military was a way to secure a job in the future:

…and the recruiter convinced me he’s like, you know, if you do computers, when you get out, you can make all this money that that’s the world. The world is IT and computers, and you can go overseas and become a contractor, make money. And so, at the time, I did not really know what I wanted to do. I just know I needed security and a job, and I did it.

Drake emphasized the hope that joining the military would open new possibilities that he currently did not have:

And so, then I got a job after college. It was worthless. And I thought, well, I’ll go in the military. I'll just talk to somebody, they said, oh, you're a college graduate. You won't have any trouble going in. And automatically you'll get to be, you know, once they see your intelligence level, they’ll certainly want you to be an officer. And I thought, oh, well, that would probably be pretty good.

As accessibility, opportunity, life stressors, conditioning, and cognitive reinforcements are components in the pathways into GD, one participant described his belief that joining the military could change his trajectory and help him escape the pathway that led him into his GD:

It kind of enabled me to kind of figure out what I want to do career-wise. You know, get a head start um like I said, I had gone through a divorce, which gambling had a huge hand in that too. And so. *When I joined the military it was like a reset button for me that I really needed*. I couldn't find it anywhere else really. (Maria, 50, female veteran, multi-racial, recovered).

### The broken promise: the military as a multidimensional risk for developing GD

When describing their pathway into gambling, either for the first time or deeper into the addiction, many veterans attributed personal characteristics, stressors, and environmental factors within their military service to their gambling addiction. Doug (59, male veteran, White, non-recovered) summarized this intersection of personal traits and the military environment as the core reason for his GD:

I brought it all on myself. I say that, but there were so many factors that contributed to me bringing it on to myself. I think that had I not joined the military, would I be a compulsive gambler? Probably not. But because I joined and all the stress, different experiences that I've had and just different people I met, and it happened turned out that way.

This segment captures the cumulative risk burden associated with military service and highlights the social and environmental factors that reinforce gambling behaviors within this context. Military-related factors function not only as independent stressors but also as mechanisms that shape ongoing learning processes and thereby contribute to the development of GD.

As in the previous theme, the participants recognized the significance of availability and accessibility to gambling during military service as a main gateway into GD or sustaining it (Blaszczynski and Nower, 2002). As shown in the literature, for participants who experienced gambling-related difficulties prior to military service, being stationed in environments with high accessibility to gambling opportunities exacerbated these problems (Dighton et al., 2025). Such contexts functioned as reinforcing environments, sustaining and further entrenching pre-existing habitual gambling behaviors. For example, Sherry (39, female veteran, Black, non-recovered) said: “Unfortunately, I got stationed right after that. That first tour to Korea to Las Vegas to Nellis Air Force Base. *So, I got it; a gambling addiction and then they sent me to Las Vegas*.”

It is interesting to note that some participants addressed the gap between the military’s official policy on gambling and the actual physical accessibility for gambling while serving, and how it affected them. While the Pathway Model situates ecological influences within broader policy environments and social acceptability, the present accounts underscore the more proximal role of physical availability as a key driver in the development of GD. This can be seen through Nate (59, male veteran, White, non-recovered): “You know, the military does have a-a rule about not gambling. It’s against the rules of the military. Although they have slot machines at some posts overseas. Not here in the US, I don’t believe, but overseas they do”.

Military service presented stressors that created a vulnerable base through which some participants described their pathway into GD. To begin with, most of the participants joined the military at a very young age (i.e., as teenagers or young adults). Entering the military at a young age acts as a risk factor for GD (Afifi et al., 2010; Tran et al., 2024). This can be understood by the fact that, though not children anymore, being exposed to difficult experiences at this age acts as a disturbance on its own, especially among those who lack coping mechanisms. An example of how being young and away from home left the participants vulnerable can be seen through Madison (42, female veteran, White, non-recovered) words:

I mean, if you think about it, the kid is 17, 18, 19-years-old, they're leaving home for the first time, they're going to a place that they haven't, you know, they don't really know what they're getting their self into, they're not fully developed, and they go through some pretty big life experiences, that could be traumatic in itself.

Another example of the intersection between young age and the risks presented through military service can be seen through Mason’s (55, male veteran, Latino, non-recovered) words:

You’re 18, you're doing this and first port that we get into, all hell’s gonna break loose. And…everything's in excess from, you know, the women to-to gambling to…um yeah, I mean, you're gonna fill that time and do it with things that the sense of um… and you only live once.

This represents the intersection of the risk-taking personality trait and the accessibility presented by military service. Research applying the Pathways Model to gambling among teens and younger individuals found that a combination of risk-taking behavior, which is common among this population, and the availability and accessibility of gambling are correlated with gambling behavior (Lee and Lee, 2025).

A major contributor to the need to gamble was intense, traumatic experiences during military service (Grubbs et al., 2023). These traumatic experiences left participants feeling exposed, vulnerable, and in need of something to help them escape these feelings, acting as a factor in their pathway to GD. Interpreted through the lens of the Pathways Model, these traumatic experiences created emotional vulnerabilities, while gambling served as a means of emotional escape (Blaszczynski and Nower, 2002; Kurilla, 2021; Nower et al., 2022b). Cody (29, male veteran, Black, non-recovered) describes this exact process:

I think, you know, people, uh gotten hurt and uh people gotten injured like terrible injuries. Um I've seen people, have been burned, you know. That's not like uh beautiful things you see like they kind of don't get uh, out of your head. So yeah so such things like, you know, they, they affect you mentally [….] You know, the thrill of winning, of gambling like… I felt stressed and overwhelmed and gambling was like one of the only things that, uh could make me happy at the moment.

Traumatic stressors did not only accumulate as a direct result of specific military jobs, but also through exposure to sexual and violent assaults during service. A systematic review on sexual harassment and trauma in military service consistently associates these experiences with an elevated risk of engaging in addictive and risky behaviors (Forkus et al., 2021). This can be understood through the same process of vulnerability and an attempt to cope through gambling behaviors, creating the illusion of relief through maladaptive gambling. An example of this can be seen through Claire (56, female veteran, Black, non-recovered), who described how she was assaulted during her military service and connected that assault to her gambling problem.

[…] there was a guy in that room with me […] I was on that table leaning against it and he jumped up on-off of his chair and bent me over onto that table and was on top of me […] I was 19 years old and I didn't have a chance to do anything[…] I think that's probably one of the reasons why I started gambling. I think… I can probably list that as why I started gambling and why it became a problem for me.

Though problematic in retrospect, the participants described the benefits of gambling during military service. For example, in contrast to the way they felt while doing their job, gambling provided them with excitement and a sense of control. Kaleb (37, male veteran, White, recovered) explained how he started gambling to cope with the long hours of his military job, which often involved shifts lasting over 10 h a day:

I think the reason why it got so bad in the military is a lot of we work 12s, and we had, we had low manning, so we were always working. We very rarely had a day off […] it was [betting], you know, something during your shift to speed the time up. You're watching the game, you got money on it, and you know, so it was just fun and exciting, and then it just turned into addicting.

Gambling also served as a way to cope with negative feelings regarding military service, including fear and loneliness (Champion et al., 2025; Dighton et al., 2025). Sherry (39, female veteran, Black, non-recovered) described the connection between gambling and stress stemming from loneliness and negative feelings about her service:

Um it can get lonely, you know, you lean on your coworkers and just the other people that are there, but I think you're still kinda separated, you know, and I think at the time, I didn't like the job […] I hated it […] I probably needed help and didn't know it. And was, and the gambling being like, you know, coping mechanism

While being or becoming more vulnerable during their military service, the participants described how the military environment and culture enhanced or even sometimes encouraged their gambling addiction (Champion et al., 2025; Vana et al., 2025). This occurred not only through accessibility and availability, as mentioned earlier, but also through military culture and comraderie. These factors increased both the habitual aspects of gambling and the conditional learning process through the acceptability of gambling and the psychological relief associated with it (Blaszczynski and Nower, 2002; Nower et al., 2022b). Many participants noted how gambling is embedded in the military socio-culture as a way to “let out steam” or bond with other soldiers. This was found in various past research exploring both pathways into GD and the prevalence of gambling in military settings (Champion et al., 2025; Paterson et al., 2021; Vana et al., 2025):

I think as a group, the military kind of doesn't have an issue with the people gambling, so they kind of take it also as a way of bonding, as a way of letting steam out. So, I think uh it's a culture that's embedded in the force (Elliot, 28, male service member, Black, non-recovered).

As with family culture, this acceptability could be interpreted as encouraging problematic gambling behaviors through positive social reinforcement (positive conditioning through the reward of feeling socially connected). Previous research on gambling motivations indicated that for some, especially individuals with social deficits, gambling becomes a way to forge social connections with others (Floyd et al., 2025). Kaleb (37, male veteran, White, recovered) describes how gambling served as a means to establish social connections in an environment that is constantly changing:

I mean it definitely helped, you know, cause you get stationed with all these people you don't know […]. If you don't know anybody, and but you know sports […] so then you're a part of the crowd, and you know, then they're texting you the next day, hey, you won, who, you who you like today, so you're instantly just jumped into, you know the circle of people that you're tight with, um, and for somebody who had already had an interest in gambling.

While for most participants, gambling problems started or worsened during their military service, six participants described how active duty also included factors that helped them manage, reduce, or control their gambling. This was a less common perspective in our interviews, but it is still important to highlight.

One way the military environment was beneficial in reducing gambling was the intensity and focus required, leaving less time for gambling. The attention and focus the participants described could be understood as a positive and adaptive coping mechanism for the stressful demands of military service. This can act as a protector, which, according to the Pathways Model, people with GD sometimes lack. For example, Elliot (28, male service member, Black, non-recovered) said that being busy in the military, and doing a job that required attention and concentration, helped reduce gambling: “Yeah. And it says during the deployment, it’s usually serious business. […] So, during those deployments, we couldn’t kind of engage in any form of gambling.”

The availability or accessibility of gambling, which sometimes diminished while being deployed overseas, was also mentioned. For example, David (45, male veteran, White, recovered) said: “if you go on assignment, you are, you are either overseas, you are in a different location, and so… you have a gambling issue and you are overseas or something. It’s just not available anymore, so“. Accessibility and availability are core components of the Pathways Model; without these components, problematic gambling cannot start or continue.

One of the participants also addressed this issue through his social connections, describing how more experienced fellow soldiers showed him ways to control his gambling, similar to self-help peer group support (Gavriel-Fried and Lev-El, 2020). “Yeah, some of the older guys who had been there for a while suggested it [ways to control the amount of money spent]. So, we kind of had, like a kind of mentor sort of thing.” (Jack, 38, male veteran, Asian, non-recovered). Through this example, it is understood that positive role models can encourage a different, more controlled form of social learning and change gambling behavior (Penfold and Ogden, 2024).

It is noteworthy that the same factors implicated in the development and maintenance of GD—namely ecological, individual, and social influences—were also identified as mechanisms supporting recovery. This apparent dual role can be understood within an ecological and behavioral framework, where risk and protective factors operate along the same continua. Research on recovery from GD suggests that engagement in structured activities, goal-directed behavior, reduced environmental accessibility to gambling, and peer support can facilitate reductions in gambling behaviors (Gavriel-Fried and Lev-El, 2020).

### Sinking deeper into gambling in the post-military period

This theme addressed the personal vulnerability that interacted with environmental factors contributing to GD after military service. The transition into civilian life is a stressful event that contributes to the participant’s sense of vulnerability (Sachdev and Dixit, 2024; Silberman, 2025), potentially worsening their gambling behaviors (Capurso, 2017; Metcalf et al., 2022).

Entering civilian life involves adjusting to a new lifestyle, where one must manage multiple challenges such as finding a job or handling finances. For individuals who lack problem-solving skills and have developed maladaptive coping strategies due to past vulnerabilities, these factors lead to greater stress and, consequently, a heightened vulnerability to GD. Cody (29, male veteran, Black, non-recovered) shared how he had to adjust to a new “lifestyle” after being discharged from the military, which, in turn, exacerbated his gambling problems:

So adjust to a new lifestyle because I've been used to the active military like I have to do this, everything is planned for. Like yeah, so I'm just there by myself, out to like adjust the new list of, maybe find out a new job, things like that. So it was kind of stressful. I think uh sometimes, you know, oh, of making money, while I'm gambling, […] that made me feel at least good and considering the other things I was going through so yeah, I say stress might have been a contributor to, my problem.

Interestingly, the participants did not elaborate on the meaning they attributed to availability and accessibility as much as in the previous themes. It is possible that this is because, at this point in their lives, they could control their own environmental factors, such as where they lived or their potential accessibility to gambling opportunities, unlike in childhood or during active service. Some participants did address the issue of environmental availability through the income they received as veterans. While this disability income could support them, research has found that veterans are more likely than others to use this money for gambling (Elbogen et al., 2024). Kaleb (37, male veteran, White, recovered) shared:

I think that's one of the things that a lot of military members like, and that contributes to gambling, as you know, on the first and the fifteenth the exact cent how much money you're getting.

Traumatic events from military service and post-trauma are major contributors to GD or to the pathway leading to GD (Sachdev and Dixit, 2024; Silberman, 2025). These were present in some of the participants’ stories, like Jared (33, male veteran, multi-racial, non-recovered), who directly connected his gambling problem to the feeling of vulnerability due to the PTSD he suffers from, which is related to his army service:

So what happens is like when you come out of the military, you-you come out not the same person you get me? You-you come out with more pain, with more damage, with trauma, and for me, I guess-for me, I must have thought gambling was a way to escape from-from reality. […] I'm completely distracting myself from the real issue, you-which is trauma.

After discharge, some veterans feel a lack of support from their close environment. This has to do with the gap experienced in the transition from belonging to a tight organization to being without it (Metcalf et al., 2023; Sachdev and Dixit, 2024; Silberman, 2025). This is especially true among veterans suffering from PTSD (Silberman, 2025). The lack of support, and thereby lack of opportunity for better coping skills—particularly in dealing with PTSD and other consequences of trauma—was described by Jared (33, male veteran, multiracial, non-recovered): “And so there aren’t that many resources, you know, like at all to help veterans; they just slap me on the back and say, ‘Good job,’ and then I’m here in Vegas.”

The Pathways Model and subsequent research confirming its main assumptions suggest that part of the addiction process involves positive reinforcement through feelings of excitement and the continuous need to engage in the exciting behavior (Blaszczynski and Nower, 2002). This process exists in other behaviors, such as extreme and adrenaline-filled experiences during military service. The transition from a life filled with adrenaline and "rush" during the active duty period to civilian life can leave veterans with symptoms similar to withdrawal (Jackson-Campos, 2025). The rush of gambling, either learned during service or after as veterans, acts as a replacement and continues as part of the conditioning process as described in the Pathways Model (Blaszczynski and Nower, 2002; Nower et al., 2022b). Some participants attributed their gambling problem as veterans to their need to feel the same rush, adrenaline, and excitement they experienced in the military (Jackson-Campos, 2025). Mark (51, male veteran, White, non-recovered), for example, shared how gambling serves as a kind of replacement for this "rush":

Like when we're in military, we’re really active. And then when you get out. There's like you hit a flat spot in your life, you know [….] I guarantee that's why I ride motorcycles and do stimulus and stuff like that. And like I said, I mean all that is taking the backseat to gambling because it's such a quick rush up and down.

Similar to elements emphasized by participants during childhood and active military service, the role of social gambling remained salient at this stage. However, rather than primarily reflecting social learning processes, gambling appeared to function as a mechanism for social connection, belonging, and informal peer support. From a social identity and belongingness perspective, these interactions may allow veterans to re-establish a sense of camaraderie and group cohesion reminiscent of their military service (Luchsinger, 2016). “Uh, usually, yeah. I usually met them at the casino we’ll be wearing our little hats, or you know, something that was and uh, and so it was kind of a camaraderie, like something that we had in common.” (Grace, 53, female veteran, Multiracial, non-recovered).

Some participants emphasized the combination of feeling vulnerable and being part of a gambling socio-cultural environment. This environment offered access to gambling while providing a way to cope with previous military or post-military vulnerabilities, which were alleviated by the gambling itself and encouraged by feelings of camaraderie and belonging. This process reinforces the addiction cycle. Nate (59, male veteran, White, non-recovered) shared how he felt the need to distance himself from others but found some social connection while going to the casino:

Kind of hard to… so I guess mainly when- when I got out um…There was no, I–I don't, I don't have relationships with people. I don't, I don't uh associate with others much. Kind of stick to myself. I'm afraid to be around people. Basically…not afraid, but I just prefer to keep my distance uh and that was because of things that occurred. But when I go to a casino now…uh at least there I can actually associate. I can talk to the person next to you with no problem.

## Discussion

This qualitative study explored the specific risk factors that shape the path leading U.S. service members and veterans to GD and its persistence over time. Our findings corresponds with the general idea of the Pathways Model for Problem and Pathological Gambling (Blaszczynski and Nower, 2002; Nower et al., 2022b), which emphasizes the interaction between individual vulnerabilities and environmental factors, and align specifically with the determinants of the second path “emotionally vulnerability problem gamblers.” This pathway encompasses ecological determinants such as accessibility and availability of slot machines on military bases, as well as early exposure in the family. It mainly includes emotional vulnerability (e.g., childhood adversity, loneliness) and life stressors before, during, and after military service, contributing to the use of gambling as a means of escaping negative affective states and life situations.

Beyond these determinants, the three themes, organized chronologically from childhood and young adulthood, through the military period, to the transition into civilian life, show how in each period the interaction between individuals’ vulnerabilities and the socio-cultural environmental contexts—including the army—manifests differently, and how transitions and turning points constitute risk factors that accelerate both the development and persistence of GD. This constitutes a unique trajectory to GD across their lifespan among U.S. veterans.

We propose expanding the Pathways Models (illustrated on the second path identified in the current study) by incorporating a life-course developmental perspective (Elder, 1994; Elder et al., 2003; Hutchison, 2005). This framework will enrich the understanding of GD trajectory with a more nuanced perspective, highlighting the cumulative process of life events and transitions. Previous longitudinal studies that explored the Pathways Models across multiple developmental periods—for example, Allami et al. (2017), who focused on adolescents and early adulthood of at-risk or problem gambling participants—were all quantitative studies that failed to capture the socio-cultural contexts in which these changes occurred, and specifically, how these risk determinants evolved and interacted over time in response to life transitions and changing social and cultural contexts.

The life-course perspective highlights how human behavior and life trajectories are shaped by personal biography, human agency (individual choices, resources, and actions), and broader social, economic, cultural, and historical contexts across the lifespan (Elder, 1994; Elder et al., 2003; Hutchison, 2010; MacLean and Elder, 2007). A core element of this life-course perspective is the role of time—specifically, how early exposure to risk factors and life conditions later influences outcomes (Elder and Giele, 2009). Trajectories represent long-term patterns of development in a person’s life, such as career paths and addictive behaviors. These trajectories are composed of transitions in roles and statuses across the lifespan (i.e., birth, marriages, divorce) and life events and turning points—significant occurrences marked by relatively abrupt changes that can generate major and lasting consequences across an individual’s life course (Hutchison, 2005). Transitions are influenced by time, age, and environmental context. Trajectories often involve several turning points, some of which may divert individuals away from their expected path, while others help redirect them toward the desired path (Hutchison, 2005; Wheaton and Gotlib, 1997).

In our findings, the transitions are related to shifts between civilian life and military service, while life events and turning points refer to stressful and traumatic events. Specifically, the first theme represents the starting point of the GD life trajectory for some participants. Adverse and traumatic events served as turning points that increased vulnerability to GD later in life. Participants’ exposure to gambling through family members and cultural norms exemplified the concept of linked lives (interpersonal and environmental networks), embedding gambling behaviors in their personal histories (Elder, 1998; Elder and Shanahan, 2007). This corresponds with two previous gambling-related studies applying a life-course perspective (Breen and Hing, 2014; Luo, 2021). For example, an exploratory, life-course perspective-based qualitative study on gambling among older Chinese people in Canada found that early years were marked by elements of loss and exposure to gambling through family traditions and behaviors. One of the key findings of this research emphasizes the element of linked lives during the participants’ childhood, establishing a connection to gambling early in life through familial ties (Luo, 2021).

The life-course perspective also acknowledges a person’s agency—the capacity to make choices—while also recognizing that such agency is constrained by environmental and relational factors (Elder, 1998). The participants’ decision to enlist in the military was described as a potential turning point and an opportunity to create a better future or even as a way to cope with preexisting GD. Past research on motivations to join the U.S. military indicated that future education, financial stability, and opportunities were among the pathways motivating individuals to enlist (Woodruff et al., 2006, 2025). This decision reflects an act of agency (MacLean and Elder, 2007), though a question arises about how the participants’ past experiences and environmental opportunities affected this choice. As mentioned above, agency is never exercised in a vacuum (Elder, 1994; Hutchison, 2010). Thus, for some participants, enlisting in the military, while meaningful, did not ultimately delay their GD trajectory, given their underlying vulnerabilities and motivations.

The second theme highlights how military service often functioned as a negative transition in their GD trajectory. The young age of participants and the stresses of military service increased their vulnerability to using gambling as a coping mechanism. According to the life-course perspective, different experiences have varying effects depending on the age at which they occur (George, 2013). The participants also shared that being young and unprepared, coupled with experiencing traumatic events while serving in the military, left them vulnerable to gambling. Military service offered new opportunities for gambling, but for those who experienced traumatic life events, it heightened the need to use gambling as a coping mechanism. For some participants, the turning point occurred before they enlisted in the military, while others described a turning point when they were offered the chance to gamble during service, with stress and trauma promoting this behavior. This finding aligns with previous research linking stress and trauma from military service to GD (Etuk et al., 2020; Metcalf et al., 2022). Similarly, a study of 1,252 U.S. Army soldiers returning from Iraq (Killgore et al., 2008) found that greater exposure to combat experiences was predictive of engagement in risk-related behaviors.

The life-course perspective highlights how individual experiences are shaped by historical periods and specific geographic contexts (Wilmoth and London, 2013). In line with this perspective, the findings showed that the location of certain military bases, given their close proximity to casinos and other venues, often facilitated gambling behaviors, which in turn played a crucial role in the development of GD among participants.

The military functions as a complex network of interconnected locations, each shaped by social organization and evolving cultural norms (Wilmoth and London, 2013). Participants also described how their lives as soldiers were linked both to fellow service members and to the general military gambling culture. For some participants, this connection between gambling and social networks originated in childhood, while for others, it first emerged during their military service. The idea of linked lives refers not only to how cultural social and peer influences shape life trajectories (Breen and Hing, 2014), but also to their impact on an individual’s agency and ability to make choices (Luo, 2021). Gambling was often perceived as a social activity that fostered belonging and alleviated loneliness, further reinforcing its cultural and social embeddedness within U.S. military culture.

The third theme represents another potential turning point in the participants’ GD life trajectory. Entering civilian life was expected to increase their agency and ability to choose and change their trajectory, as it reflected an opportunity to change their environment. As mentioned above, agency is limited to the alternatives people can perceive, which are embedded in social and structural constraints (Elder et al., 2003). Tragically, for the participants of this study, their agency in choosing to change their GD trajectory was limited by traumatic experiences from military service and a lack of support from the military institution. Furthermore, the GD was exacerbated by an environment that influenced their ability to make changes. The participants described a socio-cultural environment of gambling, even among veterans. The concept of linked lives again played a role, as veterans’ social circles often revolved around gambling, reinforcing the disorder rather than alleviating it (George, 2013). This contrasts with recent syntheses of longitudinal research indicating that PrG is generally not chronic, with most individuals classified as problem gamblers reducing their symptoms to subclinical levels or lower over time (Williams and Williams, 2025). Thus, our findings show how individual vulnerability accumulated from previous life periods interacts with socio-cultural context and new transitions, contributing not only to GD development but also to its persistence and even escalation.

Drawing on the life-course perspective, the findings of this study highlight the unique trajectories of veterans and service members, their transitions, and the turning points that led them to develop and sustain GD. It emphasizes the dynamic interplay between an individual’s personal history, their life choices regarding military enlistment and gambling engagement, and the influence of the socio-cultural environment in which they are embedded throughout their lives. The life-course approach aligns with the Pathways Model of PrG (Nower et al., 2022b) by emphasizing not only the interaction between personal and environmental factors but also how these interactions evolve over time through life transitions and turning points.

Hence, the unique contribution of this study lies in its potential extension of the Pathways Model (Blaszczynski and Nower, 2002; Nower et al., 2022b), where varied ecological and individual determinants interact with one another over time to incorporate a life-course lens. Specifically, theoretical concepts such as individual agency, life transitions/turning points, and socio-cultural contexts might offer a broader understanding of the dynamics shaping the paths into GD. Within these contexts, the manifestation of risk factors and behaviors is influenced by temporal and socio-cultural factors, as well as individuals’ subjective positioning and agency.

While our findings are broadly consistent with the Pathways Model - specifically the second subtype - they point to a more complex and continuous trajectory into problematic gambling among veterans. Specifically, our results highlight how this trajectory is shaped by the ongoing interaction between early-life vulnerabilities, experiences during military service, and the challenges associated with the transition to civilian life.

In the findings, these experiences were presented as distinct chronological stages, each reflecting elements of the Pathways Model. However, we argue that understanding these stages in isolation is insufficient. Instead, our findings suggest that these periods are dynamically interconnected, forming a cumulative and evolving trajectory rather than a series of discrete steps.

By incorporating a life-course perspective and the Pathways Model’s ideas on interactions between ecological and personal determinants, we extend our understanding of the GD trajectory of veterans. First, we foreground the role of time, demonstrating how risk factors are not static but accumulate and interact across the lifespan. Second, we highlight how the relationship between individual vulnerabilities and environmental contexts is continuously reshaped at each life stage, producing both continuity and reinforcement in gambling behavior. Importantly, this perspective helps explain not only the development of GD but also its persistence. Turning points that might otherwise represent opportunities for change—such as military enlistment or the transition to civilian life—do not necessarily disrupt the trajectory. Instead, due to the influence of linked lives (e.g., family, military peers, and veteran social networks) and constrained agency, these moments can reproduce and reinforce existing patterns of behavior. Taken together, our findings suggest a comprehensive framework for understanding veterans’ gambling trajectories—one that captures both the accumulation of risk over time and the processes that sustain engagement in gambling across different life contexts.

### Limitations

This study has several limitations: (a) The findings are based on a secondary analysis of a previous study (Vana et al., 2025); therefore, participants were not directly asked about the pathways leading to their GD. This may explain why the risk factors identified in the current study most closely resemble the second subtype of the Pathways Model of PG. However, we did not identify the key characteristics typically associated with the other two pathways. Future research should further investigate the pathways model among veterans and examine these pathways more directly. (b) Although the themes are rich in description and supported by extensive quotations, the sample includes a relatively large group of 28 military veterans. Since this is a secondary data analysis, it does not allow for saturation in its classical sense (Cheltenham et al., 2021). Moreover, we did not ask, nor did participants voluntarily mention, any cognitive beliefs (e.g., irrational beliefs, illusion of control) related to gambling. This might be partly because the initial study did not directly prompt participants to discuss their own pathways for GD; instead, they were encouraged to address external factors related to their GD (e.g., military culture, environment) and not describe the evolution of their own GD over time, which we suspect would have included examples of maladaptive cognitive beliefs. (c) Recall bias and retrospective narrative reconstruction are inherent limitations when making “pathway” claims, as participants’ accounts of past experiences may be shaped by current understandings and meanings. (d) Participants varied in age, service duration, and service experiences. Although the analysis identified common patterns across participants, future research should examine how age and length of military service influence gambling pathways. (e) Our sample comprised only eight women, and we recommend future research explore the unique pathways relevant to women. (f) Because the life-course perspective emphasizes historical and contemporary social-cultural contexts, variations in service era could not be fully explored, which also highlights an area for future research. (g) The study may be subject to self-selection bias, as participants who were willing to be interviewed—recruited via flyers and social media—may differ from those who did not volunteer, potentially leading to an overrepresentation of individuals with more severe or salient experiences. (h) An additional potential limitation may relate to the online (Zoom) modality used for qualitative interviews, which could have influenced participants’ willingness to disclose sensitive information, potentially affecting openness in ways that are not fully clear. However, prior research suggests that key indicators of interview quality do not substantially differ between face-to-face and online modalities (Peasgood et al., 2023). (i) Finally, although the sample included veterans and service members from diverse military branches, service eras, and lived experiences, key service-related variables were not systematically examined. While some participants described gambling in the context of deployment, the study did not explicitly assess the impact of deployment history (combat vs. noncombat), length of military service, or era of service on GD. As such, the findings should be interpreted with caution, as these factors may significantly influence one’s pathway to GD. Future research should more directly evaluate these variables to better delineate their role in the development and maintenance of GD among military populations.

#### Recommendations for practice and for future studies

The findings highlight how the socio-cultural environment within the U.S. military contributed to the development of GD among service members and veterans. This has important policy implications. For instance, U.S. military leadership should reduce access to gambling opportunities on military bases (i.e., by removing on-base slot machines) or increase access for service members to non-gambling wellness activities on and off the base. Alternatively, military leadership could also consider educating service members on responsible gambling strategies (e.g., setting limits on the amount of time or money spent, avoiding gambling for emotional reasons such as boredom, sadness, or stress, and not chasing gambling losses) as a means of minimizing harm. Prior research suggests that promoting self-regulatory strategies is effective in reducing gambling harms (Hing et al., 2024).

Greater awareness efforts within the U.S. military are recommended to promote early screening, routine assessment (i.e., conducted annually), and timely intervention. Expanding access to on-base treatment and peer-led gambling support programs may be particularly beneficial for service members who enter the military with preexisting gambling problems or who develop problem gambling during their service, especially when deployed overseas and working in a highly stressful environment. Post-discharge, comprehensive support should address PTSD, employment, financial literacy (including gambling literacy), social integration, and emotional wellbeing to reduce vulnerability during the transition to civilian life and to prevent maladaptive coping behaviors, including gambling.

## Data Availability

The raw data supporting the conclusions of this article will be made available by the authors, without undue reservation.
